# Resilience as a Predictor of Indirect Trauma Among Korean Adolescents: A Cross-Sectional Correlational Study

**DOI:** 10.3390/healthcare13192491

**Published:** 2025-09-30

**Authors:** Suyon Baek

**Affiliations:** 1 Department of Nursing, College of Nursing and Health, Kongju National University, Gongju-si 32588, Republic of Korea; whitesy@kongju.ac.kr; Tel.: +82-41-850-0303; 2 Nursing Convergence Research Center of Kongju National University, Gongju-si 32588, Republic of Korea

**Keywords:** adolescent, disaster, psychological trauma, resilience, social support

## Abstract

**Background/Objectives:** Adolescents aged 13–18 are exposed to traumatic content even without direct experience, owing to the increasing media coverage of disasters. Such indirect exposure can result in post-traumatic stress symptoms, including intrusion, avoidance, and hyperarousal, as well as associated emotions such as sadness, anger, and guilt. These effects may persist for months, reflecting the vulnerability of adolescents during cognitive and emotional development. This study examined resilience and social support as protective predictors against indirect trauma. **Methods:** A cross-sectional correlational design was employed, with middle- and high-school students aged 13–18 years in Seoul, South Korea, as participants. Indirect trauma, resilience, and perceived social support were assessed using validated self-report instruments. Correlation analyses were conducted, followed by stepwise regression. Owing to multicollinearity, resilience was retained as the sole predictor in the final model. **Results:** The average indirect trauma score was 1.20 out of 4, and 59.2% of participants exhibited partial or full post-traumatic stress disorder. The mean resilience and social support scores were 3.47 and 3.82 out of 5, respectively. Resilience was positively correlated with social support (r = 0.60, *p* = 0.001). The regression analysis indicated that resilience significantly predicted indirect trauma (β = 0.82, *p* < 0.001), accounting for 66.4% of the variance, whereas social support showed no direct effect. **Conclusions:** Resilience emerged as a key predictor of indirect trauma, underscoring its importance in mitigating distress. Although social support did not directly predict trauma, its positive correlation with resilience suggests potential indirect effects. These findings highlight the need to strengthen resilience and expand school-based counseling and support systems to help adolescents deal with indirect trauma.

## 1. Introduction

A disaster is an event in which natural or man-made hazards are combined with the vulnerabilities of human society to cause serious disruption to societal functioning [1]. Various disasters occur domestically and internationally on a regular basis. With the development of the Internet and social networking services (SNSs), information related to these disasters is disseminated to numerous people globally at a rapid pace, without being filtered. Studies have confirmed that such indirect trauma experiences can cause post-traumatic stress symptoms (PTSSs), including intrusion, avoidance, and hyperarousal, as well as related negative emotional responses [2,3,4,5,6].

Indirect trauma refers to psychological distress experienced by individuals who are not directly exposed to a traumatic event but are affected secondarily through media coverage, stories, or relationships with victims [7]. Studies on indirect trauma have focused on concepts such as secondary and vicarious trauma, and most studies have targeted occupational groups (e.g., police officers, counselors, and social workers) that directly support trauma victims [8,9,10].

The Sewol Ferry disaster that occurred in Korea in 2014 was a large-scale traumatic event that caused indirect trauma to numerous people. Research has gradually expanded beyond studying specific occupational groups to include other individuals. A study targeting 346 middle- and high-school students who indirectly experienced the Sewol Ferry disaster reported that approximately 72% of the participants exhibited PTSSs, such as hyperarousal, avoidance, and intrusion [2]. They experienced diverse emotions, such as sadness, anger, depression, emptiness, and guilt. Another study surveyed 76 UK elementary school students aged 10–11 regarding their PTSSs following the 9/11 terrorist attacks in the United States. It was found that 14.5% of the students experienced moderate to severe PTSSs even after two months of the incident, and that 9.2% of them suffered from such symptoms even after six months [11]. Neurological evidence has indicated that adolescence is a period in which emotional regulation and stress response systems are not yet mature, making adolescents more vulnerable to indirect trauma [12]. Moreover, adolescents use social media and online platforms most actively and are likely to be repeatedly exposed to disaster-related images and information [13]. Therefore, from a policy perspective, adolescents should be considered a priority group that requires early screening and intervention for mental health problems in disaster situations [12], and their indirect trauma experiences should be carefully considered.

However, research examining indirect trauma in adolescents in Korea is lacking [2,14,15]. Two studies have investigated the degree of indirect trauma in adolescents as an independent variable and its effects on their mental health, such as post-traumatic stress and depression [14,15]. Only one study has explored the factors affecting the degree of indirect trauma in adolescents [2]. Several recent studies have examined predictors relating to indirect trauma and PTSSs among adolescents worldwide. The findings showed that resilience functions as a key personal characteristic that alleviates trauma responses [16], whereas family and social support serve as important external resources that promote psychological stability [17]. In addition, school counseling services and support programs help adolescents manage emotional difficulties and adapt in the aftermath of disasters [16]. The scale and extent of disaster-related damage and the level of media exposure increase the severity of indirect trauma and prolong PTSSs [16].

Based on this evidence, the present study examined the degree of indirect trauma among Korean middle- and high-school students and investigated the roles of resilience and social support, which have been identified as major influences on the indirect trauma responses of adolescents in prior research. In particular, this timely study is significant because many adolescents are expected to experience indirect trauma in Korea owing to the frequent occurrence of accidents, such as the Itaewon disaster in 2022 [18], Osong underpass flooding accident in 2023 [19], and J Airlines accident in 2024 [20]. Most existing interventions for youth in Korea and other countries have investigated direct rather than indirect trauma. However, these trauma types should be addressed separately, as their mechanisms and effects may differ [21]. In this context, the findings of the present study can provide foundational evidence for designing interventions that are specifically tailored to adolescents experiencing indirect trauma.

### Purpose

This study aimed to identify the following:

1. The general characteristics of participants.
2. The resilience, social support, and indirect trauma of participants.
3. The correlations among the resilience, social support, and indirect trauma of participants.
4. Predictors of the severity of indirect trauma among adolescents.

## 2. Methods

### 2.1. Research Design

A descriptive, cross-sectional, and correlational design was employed to examine the effects of resilience and social support on the indirect trauma experiences of Korean adolescents following disasters.

### 2.2. Research Participants

Adolescents were defined as individuals between 13 and 18 years old, corresponding to middle- and high-school students in South Korea. Participants were selected using convenience sampling from adolescents residing in Seoul Metropolitan City. The inclusion criteria were as follows: (1) between 13 and 18 years old and currently enrolled in school; (2) capable of understanding the purpose and procedures of the study; (3) provided written informed consent; and (4) obtained written consent from their legal representatives. The minimum number of participants was determined to be 118 based on a regression analysis power of 0.80, a significance level of 0.05, an effect size of 0.15 [22], and a predictor variable of 10 using the G*Power 3.1.9.4 program. Considering a dropout rate of 10%, the survey was conducted with 130 participants.

### 2.3. Instruments

#### 2.3.1. Indirect Trauma

Indirect trauma was measured using the Impact of Event Scale-Revised-Korean (IES-RK). Eun et al. [21] translated and revised the IES-R to validate the Korean version, and children and adolescents aged 10–19 years were included in the validation sample. The IES-RK comprises 22 items, including hyperarousal (six items), avoidance (eight items), and intrusion (eight items). Responses are rated on a five-point Likert scale: “not at all” (0), “a little bit” (1), “average” (2), “quite a bit” (3), and “very much” (4). A higher total score for all items indicates a more severe level of post-traumatic stress; 24/25 points is the cutoff for distinguishing complete from partial post-traumatic stress disorder (PTSD), whereas 17/18 points is the cutoff for distinguishing partial PTSD from normal [23]. The Itaewon disaster in 2022 and Osong underpass flooding incident in 2023 were used as examples of disasters when measuring indirect trauma to improve the understanding of the research participants. The reliability and validity verification of the IES-RK revealed Cronbach’s alpha = 0.87, 0.70, and 0.63 for hyperarousal, avoidance, and intrusion, respectively. Previous research with Korean adolescents reported Cronbach’s alpha = 0.885, 0.841, and 0.857, respectively, with 0.935 overall [2]. In this study, the overall reliability was Cronbach’s alpha = 0.95, with 0.87, 0.86, and 0.88 for the respective domains.

#### 2.3.2. Resilience

*Resilience* is defined as the capacity of an individual to adapt positively to and recover from adversity, stress, or trauma [24]. In this study, resilience was measured using the Resilience for Korean Adolescents tool developed by Shin et al. [25]. The tool consists of 27 items, with three items for measuring each of the domains causal analysis, emotional control, impulse control, gratitude, life satisfaction, optimism, relationships, communication skills, and empathy. Resilience for Korean Adolescents represents a critical psychological resource for the developmental stage of adolescence and is structured in alignment with the concept of resilience as defined in the existing psychological literature [24,26]. It uses a five-point Likert scale, with responses ranging from “not at all” (1) to “very much” (5), where higher scores indicate higher resilience. At the time of development of the instrument, its reliability was Cronbach’s alpha = 0.624–0.799 for each domain, and the overall reliability in this study was Cronbach’s alpha = 0.87.

#### 2.3.3. Social Support

*Social support* is defined as an individual’s subjective perception of the availability and adequacy of support received from three sources: family, friends, and significant others [27]. In this study, social support was measured using the Multidimensional Scale of Perceived Social Support (MSPSS) [27], which was validated in South Korean adolescents [28]. Previous studies have confirmed that the MSPSS captures the developmental context of adolescence, during which peer relationships gain salience, family dependence undergoes changes, and support from teachers or other adults in school environments plays an important role [29]. It consists of 12 items rated on a five-point Likert scale, with responses ranging from “not at all” (1) to “very much” (5). Higher scores indicate greater perceived social support. At the time of the Korean translation, its reliability was Cronbach’s alpha = 0.89. Previous research with Korean adolescents reported Cronbach’s alpha = 0.95 overall [28]. The overall reliability in this study was Cronbach’s alpha = 0.91.

### 2.4. Data Collection

Data were collected between 22 October and 8 November 2024. The research data collectors provided the participants with information regarding the purpose, procedures, and methods of the research, the guarantee of anonymity, and the possibility of discontinuing participation in the research at any time. The participants and their legal representatives received a consent form. Data were collected only from participants who voluntarily agreed to participate in the research. The questionnaire was filled out directly by the participants, and it took approximately 20 min to complete. After the survey was completed, the research data collectors retrieved the questionnaire and mailed it to the researchers.

### 2.5. Data Analysis

Data analysis was performed using SPSS/WIN 28.0, and the specific methods used were as follows:

1. The general characteristics, resilience, social support, and indirect trauma of the participants were analyzed using descriptive statistics such as frequency, percentage, mean, and standard deviation (SD).
2. The normality of the data was examined using the Kolmogorov–Smirnov and Shapiro–Wilk tests, and skewness and kurtosis values within ±2 were considered indicative of a normal distribution.
3. Indirect trauma, according to the general characteristics of the participants, was analyzed using a t-test and analysis of variance (ANOVA), and post hoc analysis was performed using the Scheffé test.
4. The correlations among the resilience, social support, and indirect trauma of the participants were analyzed using Pearson’s correlation.
5. Factors affecting the indirect trauma experienced by the participants were analyzed using multiple regression.

### 2.6. Ethical Considerations

This study was approved by the Institutional Review Board of Kongju National University (KNU_IRB_2024-083). This was a questionnaire-based study, and the explanatory documents provided sufficient information regarding the research. Participation in the study was voluntary; there were no disadvantages to refusing to participate, and participants could withdraw at any time if they wished. The data collected were used only for research purposes. All participants who responded to the questionnaire received small gifts. Research-related data will be kept for three years after the end of the study and then destroyed using a shredder.

## 3. Results

### 3.1. General Characteristics and Corresponding Indirect Trauma of Participants

Most participants were male students (55.4%), and the largest proportion was 16 years old (23.8%). Among the participants, 50.5% responded that their satisfaction with school life was high. The main mode for obtaining information regarding disasters was the Internet or social media (75.4%). The experience rate of post-disaster education programs was 82.3%, that of emotional support programs following a disaster was 43.1%, and that of counseling with teachers following a disaster was 15.4%. Among the participants, 74.6% responded that education or counseling programs regarding disasters are needed. The differences in indirect trauma according to the general characteristics of the participants were not statistically significant (Table 1).

### 3.2. Resilience, Social Support, and Degree of Indirect Trauma of Participants

The resilience level of the participants was 3.47 ± 0.55 out of 5 points, and the social support level was 3.82 ± 0.77 out of 5 points. The indirect trauma level of the participants was 1.20 ± 1.14 out of 4 points. Among the 130 participants, 40.8% were classified as having no PTSD, 9.2% as having partial PTSD, and 50.0% as having full PTSD (Table 2).

### 3.3. Correlations Among Resilience, Social Support, and Indirect Trauma of Participants

Neither resilience and indirect trauma nor social support and indirect trauma were significantly correlated. However, resilience and social support exhibited a significant positive correlation (r = 0.60, *p* = 0.001) (Table 3).

### 3.4. Predictors of Indirect Trauma of Participants

In this study, general demographic variables such as gender, age, and previous program experience were not included in the regression analysis. This decision was based on preliminary statistical tests (independent t-tests and one-way ANOVA), which revealed no significant differences in the dependent variable (indirect trauma) across these characteristics. Therefore, including these variables was deemed unnecessary as they did not contribute meaningfully to the explanatory power of the model. This approach is consistent with the principle of model parsimony, which recommends excluding non-contributory predictors to avoid overfitting and enhance interpretability [30].

Neither resilience nor social support showed a significant simple correlation with indirect trauma. However, as correlation and regression analyses serve different purposes, a stepwise regression analysis was conducted to estimate the unique contribution of each predictor while controlling for overlapping variance [31,32].

In the stepwise multiple regression, resilience entered at Step 1 and remained the sole predictor; social support did not meet the entry criterion and was excluded because of multicollinearity. The final model was significant, with R = 0.817, R^2^ = 0.667, adjusted R^2^ = 0.664, SEE = 18.55, F (1,129) = 258.15, *p* < 0.001, and Durbin–Watson = 1.705. Resilience showed a significant unique effect on indirect trauma (B = 0.276, SE = 0.017, β = 0.817, t = 16.07, and *p* < 0.001). When forced into the model, social support was non-significant (β = 0.007, t = 0.021, and *p* = 0.983) and exhibited severe collinearity (tolerance = 0.026; VIF = 38.07). These results indicate that, after accounting for overlap with social support, resilience explained 66.4% of the variance in the indirect trauma of adolescents. Therefore, adolescents with higher levels of resilience reported lower levels of indirect trauma symptoms (Table 4).

## 4. Discussion

This study aimed to investigate the degree of disaster-related indirect trauma and its associated predictors, namely resilience and social support, among 130 middle- and high-school students. Because limited research has been conducted on adolescents who have indirectly experienced natural and social disasters in Korea, the results of this study are significant as basic data for developing interventions for adolescents who have experienced indirect trauma but have not been included as targets of intervention.

The participants of this study exhibited a moderate level of indirect trauma. A substantial proportion of the participants were classified into the partial or full PTSD groups. A previous study was conducted in July 2014 following the Sewol Ferry disaster in Korea, using the same instrument and targeting 346 middle- and high-school students [2]. The average score for indirect trauma was 28.58; this was somewhat lower than the results for the partial or full PTSD groups, which included 72.3% of the students. However, this figure is higher than the results of another study conducted in October 2014 [14], which investigated the degree of indirect trauma among 244 first-year high-school students and found that 28.7% were classified into the partial or full PTSD groups. In addition, a study that investigated the prevalence of PTSSs among Chinese adolescents during the COVID-19 pandemic using the same instrument (IES-R) [17], a total score of 20 or higher was considered positive for PTSSs, which is higher than the scores of the 35.7% of adolescents who reported having experienced PTSSs. The participants in this study reported a high rate of experiencing PTSSs, probably because of the expanded influence of the Internet and SNSs since 2014 and subsequent repetition of disasters, such as the Itaewon incident and COVID-19 pandemic. However, this score was lower than that of 2.06 out of 4 reported in a study that measured the degree of indirect trauma among adolescents owing to COVID-19 using a different instrument [15]. This is because the instrument in this study measured avoidance, intrusion, and hyperarousal, which are the main symptoms of PTSD, whereas the tool used in the previous study [15] measured changes in individual cognition and emotion, the distrust response to society, and the intrusion response [33]; among these, the distrust response to society was the highest at 2.23 points.

The participants exhibited a moderate level of resilience, which was similar to that observed in foster youth [34] and elementary school students [35] when the same instrument was used. Previous studies have shown that resilience alleviates the negative effects of various environments experienced by adolescents and serves as a protective factor for their adaptation to hardships and adversity [36]. Similarly, higher resilience is associated with greater post-traumatic growth, such as in bereaved families of adult suicide victims [37]. The present study also showed that resilience has a significant effect on the indirect trauma experiences of adolescents. The findings suggest that resilience serves as a protective factor that mitigates the psychological impact of disaster-related indirect trauma. Adolescents with higher levels of resilience are likely to regulate their emotions more effectively, reframe stressful experiences, and cope adaptively with disaster-related media exposure. This interpretation is consistent with prior evidence indicating that resilience serves as a buffer against adverse psychological outcomes in adolescents [38]. However, research on the relationship between resilience and indirect trauma or indirect post-traumatic growth in adolescents is lacking. Therefore, further research is required to demonstrate their relationship. Accordingly, indirect trauma intervention programs using resilience should be developed, and effectiveness verification studies should be conducted.

At the school level, resilience-building programs can be included in the curriculum and counseling services to strengthen the coping strategies, emotional regulation, and media literacy of adolescents. Teachers and school counselors are central in fostering supportive environments in which students can express distress and receive timely assistance. Evidence from international research supports the effectiveness of such approaches. For example, Berger et al. [38] conducted a cluster randomized controlled trial in Israel and demonstrated that a universal school-based resilience program significantly reduced post-traumatic stress, anxiety, and depression among high-school students. Likewise, a systematic review by Dray et al. [39] confirmed that universal resilience interventions that were delivered in schools improved emotional well-being and mental health in diverse youth populations. These findings underscore the value of school systems as accessible and sustainable platforms for reducing the psychological impact of indirect trauma. Effective resilience programs typically include components such as emotional regulation training, cognitive restructuring, problem-solving skills, and peer-based support activities. Enhancing media literacy to address the psychological burden of repeated disaster exposure and embedding interventions within multi-tiered support structures involving teachers and counselors can further enhance their impact. Both relevance and scalability can be ensured by tailoring programs to the developmental needs of adolescents and delivering them within school settings.

At the policy level, systematic strategies are essential. These include implementing standardized school-based resilience curricula, providing specialized training for educators, and ensuring strong connections with community mental health services. Integrating such measures into national disaster preparedness and response frameworks can strengthen the capacity to safeguard vulnerable adolescents from the psychological consequences of indirect trauma.

However, in this study, resilience did not show a significant simple correlation with indirect trauma, but emerged as a strong predictor in the regression analysis. This discrepancy can be attributed to the methodological distinction between correlation and regression analyses; while correlation examines only simple linear associations between variables, regression estimates the unique effect of an independent variable on the dependent variable while controlling for the influence of other predictors [31]. In this study, the high correlation between social support and resilience led to multicollinearity [30], and consequently, social support was excluded from the final regression model. Therefore, the unique explanatory power of resilience became more apparent. These findings suggest that resilience may not operate solely as an independent predictor of indirect trauma but may instead function as a mediator or moderator within its interaction with social support. Therefore, structural equation modeling or mediation/moderation analyses should be employed in future research to clarify the underlying mechanisms and delineate the role of resilience further.

In addition, an explanatory power exceeding 50% in multiple regression analysis is considered very high in general psychology and social science research [31]. In this study, the explanatory power of the single variable of resilience was 66%, which could indicate the possibility of various biases. Common method bias and bias due to the characteristics of the research participants can be considered as limitations of this study. Common method bias refers to a systematic error that may occur when independent and dependent variables are measured simultaneously using the same method [40]. This study simultaneously measured the variables using the same method via a self-report questionnaire. In future studies, the presence of common method bias must be verified and, if necessary, controlled [41]. In terms of bias owing to the characteristics of the participants, the number of participants experiencing partial or complete PTSD was high in this study. Thus, resilience might have had a strong effect on adolescents with high levels of PTSD. However, as research on resilience and indirect trauma among adolescents is insufficient, such interpretations should be made with caution.

The participants reported a relatively high level of perceived social support, which was higher than the score of 2.17 obtained in a study that investigated 219 middle-school students with complex traumatic experiences using the same instrument [42] and the 33 out of 77 points reported in a study that investigated students using a different instrument [2]. In a study that identified factors related to indirect trauma in adolescents who experienced the Sewol Ferry disaster, social support did not have a significant influence [2]. In this regard, the author concluded that social support techniques that do not resolve the underlying problems and emotions may be maladaptive [43] and that telling negative stories related to traumatic events may have a counter-buffering effect that increases post-traumatic stress [2]. In addition, when a personal traumatic event occurs, parents, teachers, and friends provide comfort and advice and show concern, whereas support for social events is ambiguous and qualitatively different. However, it does not act as a protective factor against indirect trauma because it is a form of repeated communication that frequently highlights the negative aspects of the incident, rather than providing catharsis through the expression of emotions [44].

Nonetheless, social support has a moderating effect on the relationship between the complex trauma experiences and PTSD of students [42], and research has shown that social support from foster youths affects their subjective well-being through resilience [34]. Although social support did not emerge as a significant predictor of indirect trauma in the present study, this finding should not be interpreted as evidence of its irrelevance. Instead, it highlights the need to consider the multidimensional nature of social support. The effects of support may differ according to the type of provider (e.g., family, peers, and teachers), quality and adequacy of support (e.g., emotional versus instrumental), and context in which it is provided. Therefore, future research should distinguish among these dimensions and incorporate qualitative or mixed-method approaches to explore how adolescents perceive and utilize social support following disasters. The findings of this study may also reflect the possibility that social support is interpreted and experienced differently across developmental stages, and that the level of emotional maturity of adolescents influences both the effectiveness and perception of such support [45]. Therefore, future research should compare developmental differences and incorporate emotional regulation capacities as potential mediating or moderating variables to elucidate these mechanisms more thoroughly.

Finally, social support was significantly and positively correlated with resilience in this study. This suggests that social support may indirectly influence trauma outcomes by bolstering adolescents’ capacity for resilience. Therefore, interventions targeting the indirect trauma of adolescents should incorporate social support strategies that extend beyond emotional reassurance and instead foster cognitive and behavioral coping skills. Given the strong correlation between social support and resilience, future research should examine whether resilience serves as a mediator between social support and indirect trauma. Clarifying this indirect pathway could provide a deeper understanding of the psychological effects of social support and aid in the design of more effective trauma-informed interventions. Mediation analysis using structural equation modeling or bootstrapped indirect effect estimation is recommended to test this mechanism rigorously.

Overall, our findings indicate that interventions for adolescents experiencing indirect trauma should move beyond traditional PTSD-focused approaches. Conventional PTSD interventions have primarily emphasized trauma reprocessing and exposure-based strategies. In contrast, our results, which show resilience as the most influential factor and social support as having no direct effect, together with evidence of the developmental vulnerabilities of adolescents in emotional regulation and stress response systems [12,46], highlight the need to prioritize resilience building, coping skills, emotional regulation, and media literacy within school-based curricula. Moreover, given the significant positive correlation between resilience and social support, interventions should focus on enhancing the quality of peer and teacher support as mechanisms for strengthening resilience, rather than treating social support as a standalone protective factor.

## 5. Conclusions

This cross-sectional study investigated the degree of disaster-related indirect trauma and its associated predictors, namely resilience and social support, among 130 middle- and high-school students. The results highlight the high prevalence of stress symptoms among adolescents who are indirectly exposed to disasters, with resilience identified as a critical predictor for mitigating distress. Although social support did not directly predict indirect trauma, its strong correlation with resilience underscores its indirect importance. The results of this study provide foundational data for informing the development of such interventions.

This study has several limitations that warrant consideration. First, owing to its cross-sectional design, the causal relationships among resilience, social support, and indirect trauma could not be conclusively established. Longitudinal designs are required to clarify the temporal sequencing and potential bidirectional influences among these variables. Second, the generalizability of the findings is limited because the participants were selected exclusively from adolescents residing in Seoul, South Korea. Regional, cultural, and contextual factors, including educational environments and societal attitudes towards trauma and emotional expression, may shape both the perception of social support and the development of resilience. Therefore, caution should be exercised when extrapolating these findings to adolescents in other regions or cultural contexts. To strengthen the external validity, participants should be recruited from diverse geographical areas, and cross-cultural comparative studies must be conducted to examine the universality versus cultural specificity of these associations. Third, this study relied on self-reported measures. In future research, a multi-informant approach should be adopted by incorporating additional perspectives such as teacher or parent reports, as well as clinical assessments or structured psychological evaluations, to improve the validity and reliability of the findings. Such triangulation would allow for a more comprehensive understanding of the indirect trauma experiences of adolescents and provide stronger evidence for the development of tailored interventions.

## Figures and Tables

**Table 1:** General characteristics and indirect trauma of the participants (N = 130).

Characteristics	Categories	n (%)	Indirect Trauma	
			Mean ± SD	t or F(*p*) Scheffé
Gender	Male	72 (55.4)	27.82 ± 17.59	0.94 (0.349)
	Female	58 (44.6)	24.83 ± 18.62	
Age	13	24 (18.5)	27.46 ± 21.14	0.69 (0.633)
	14	26 (20.0)	26.73 ± 16.83	
	15	15 (11.5)	23.67 ± 24.26	
	16	31 (23.8)	28.00 ± 16.23	
	17	23 (17.7)	29.00 ± 16.64	
	18	11 (8.5)	18.09 ± 11.31	
School life satisfaction	High	65 (50.5)	27.94 ± 18.81	0.44 (0.646)
	Middle	53 (40.8)	25.25 ± 16.37	
	Low	12 (9.2)	24.08 ± 21.62	
Primary channels for obtaining disaster information	Media	17 (13.1)	32.35 ± 26.72	1.061 (0.379)
	Internet or SNS	98 (75.4)	24.73 ± 16.18	
	Parents	7 (5.4)	31.57 ± 19.57	
	Teachers	2 (1.5)	23.50 ± 14.85	
	Friends	6 (4.6)	33.50 ± 16.08	
Experience with educational programs following disasters	Yes	107 (82.3)	26.97 ± 18.51	0.663 (0.509)
	No	23 (17.7)	24.22 ± 15.86	
Experience with emotional Support programs following disasters	Yes	56 (43.1)	25.55 ± 17.06	−0.510 (0.611)
	No	74 (56.9)	27.19 ± 18.84	
Experience with counseling with teachers following disasters	Yes	20 (15.4)	31.00 ± 18.64	1.219 (0.225)
	No	110 (84.6)	25.66 ± 17.90	
Need for counseling or educational programs	Needed	97 (74.6)	26.07 ± 18.17	−0.445 (0.657)
	Not needed	33 (25.4)	27.70 ± 17.89	

Note. SNS: social networking service.

**Table 2:** Level of resilience, social support, and indirect trauma of the participants (N = 130).

Variables	Item		Total		
	Mean ± SD	Range	Mean ± SD	Range	N (%)
Resilience	3.47 ± 0.55	1–5			
Social support	3.82 ± 0.77	1–5			
Indirect trauma	1.20 ± 1.14	0–4	26.48 ± 18.05	0–88	
No PTSD	0.40 ± 0.53	0–4	8.77 ± 4.60	0–17	53 (40.8%)
Partial PTSD	0.95 ± 0.06	0–4	21.00 ± 1.28	18–24	12 (9.2%)
Full PTSD	1.91 ± 0.52	0–4	41.94 ± 11.45	25–88	65 (50.0%)

**Table 3:** Correlations among resilience, social support, and indirect trauma of the participants (N = 130).

Variables	Resilience	Social Support	Indirect Trauma
	r (*p*)	r (*p*)	r (*p*)
Resilience	1		
Social support	0.60 (0.001)	1	
Indirect trauma	−0.01 (0.925)	−0.05 (0.587)	1

**Table 4:** Stepwise regression analysis for predictors of indirect trauma (N = 130).

Variables	B	SE	β	t	*p*	Tolerance	VIF
Resilience	0.28	0.02	0.82	16.07	<0.001	1.000	1.000
Social support †			0.01	0.02	0.983	0.026	38.072
R^2^ = 0.667; adj. R^2^ = 0.664; SEE = 18.55; F (1,129) = 258.15; *p* < 0.001; Durbin–Watson = 1.705.							

Notes: Dependent variable = indirect trauma. Stepwise criteria: *p* to enter ≤ 0.050, *p* to remove ≥ 0.100. † Excluded by the stepwise algorithm; statistics shown are “if entered,” indicating severe multicollinearity.

## Data Availability

The data presented in this study are available on request from the corresponding author (The data are not publicly available due to privacy or ethical restrictions).

## Abbreviations

The following abbreviations are used in this manuscript:

SNS	Social networking service
PTSD	Post-traumatic stress disorder
IES-RK	Impact of Event Scale-Revised-Korean
PTSS	Post-traumatic stress symptom
